# Water quality assessment of Ashmaq and Sawur streams using physicochemical and biological indicators, Kewote district, Central Ethiopia

**DOI:** 10.1038/s41598-026-65963-x

**Published:** 2026-08-11

**Authors:** Alamrew Eyayu, Tsige Woldehawariat

**Affiliations:** 1 Department of Biology, Debre Tabor University, P. O. Box. 272, Debre Tabor, Ethiopia; 2 Department of Biology, Debre Berhan University, P. O. Box. 445, Debre Berhan, Ethiopia

**Keywords:** Kewot district, Physicochemical parameters, Streams, Water quality, Ecology, Ecology, Environmental sciences, Ocean sciences, Zoology

## Abstract

Tropical streams have distinct natural features that are heavily influenced by human activities, especially in developing regions. The impact of human activities is measured by exploring changes to the streams’ natural physicochemical properties and biological communities. However, information on the physicochemical and biological characteristics of smallest streams with great socioeconomical importance remains scarce in Ethiopia. Thus, this study assessed the physicochemical and biological characteristics of Ashmaq and Sawur Streams (Aug 2022–Apr 2023) to evaluate human impacts on stream ecology of the rural areas. The area of this region is generally semi-arid characterized by erratic rainfall patterns where June to August and December to April, are rainy and driest months, respectively. Water and macroinvertebrate samples were taken at two sampling sites of each stream at different water depths using sterilized plastic bottles and a D-frame net, respectively and gillnets and hooks were used to sample fish. Accordingly, a total of 1386 and 149, macroinvertebrates and fish, respectively were sampled. In the laboratory, water samples were analyzed using the American Public Health Association (APHA) standards. ANOVA was employed to show the significant difference in spatio-temporal patterns of stream water physicochemical parameters. In the present study, EC ranged between 187.90 ± 61.60 to 662.00 ± 6.56 µScm^-1^, and highest values were recorded during the wet months. Temperature, pH, dissolved oxygen, chloride, and nutrients (NO^2-^ and PO_4_^2-^) were major environmental variables that characterize distribution patterns of fish and macroinvertebrate communities. Almost all the in-situ examined physicochemical parameters revealed spatio-temporal significant differences in both streams. Corixidae was the dominant family of macroinvertebrates, while *Labeobarbus intermedius* accounted for 67.4% of the total collection of fishes identified. In conclusion, most physicochemical features of Ashmaq and Sawur streams were below the permissible limits set by international standards. However, our personal observation encountered that Ashmaq and Sawur streams are facing several ecological threats including poor agricultural practices, water abstraction, intensive grazing, and sand mining. Therefore, this baseline data on the tiny percentage of global freshwater ecosystems could help with management implications like habitat restoration and raising public awareness and educating people about the need to save water and their biodiversity.

## Introduction

Nutrient loading in terrestrial, freshwater and coastal ecosystems occurs because of human waste disposal and agriculture on a global scale. Since they are essential for the survival of various life forms, freshwater resources are major concerns of conservation^1–5^. Moreover, freshwater resources contribute only 3% of the total water resources, out of which only 1% is available for human usage^5^. Due to the population explosion and higher human dependency on freshwater sources, these ecosystems are under tremendous stress and are deteriorating at a faster rate^5,6^. The area of freshwater sources is shrinking and is also being polluted by anthropogenic influences; thus, both the quantity and quality are compromised. Today, the world is struggling with basic consumptive water usages like drinking, manufacturing, livestock rearing, and agriculture due to the dilapidated condition of inland water resources and their distribution^7^. As a cumulative effect of all these human demands, over the past decades elsewhere has been compensated by streams and rivers^5,8,9^. However, these ecosystems continue to experience deterioration in quality and quantity due to brutal human activities^5,6,10^.

In Ethiopia, the water quality of fluvial habitats is significantly impacted by pollution from urban runoff, industrial and domestic waste, and agricultural chemicals, leading to high levels of contaminants like heavy metals, bacteria, and nutrients^6,11–13^. Rivers and streams particularly located in urban agricultural areas and industrial zones, often show poor quality that is not suitable for domestic or even irrigation purposes^4,14,15^. To the extent of our knowledge, there is a lack of publicly available research specifically on the water quality of the Ashmaq and Sawur streams in Ethiopia. However, data from studies on other Ethiopian waterways, especially tributaries of the Awash River, can be used to infer potential water quality issues contaminated by agricultural runoff, untreated waste, and soil erosion^5,13,14^. Thus, widespread surface water pollution is a significant problem in Ethiopia, stemming from similar agricultural and urban factors across various regions^3,13,15,16^. Based on these and other broader Ethiopian water studies, it is almost certain the water is unsafe for drinking without treatment in many areas of Ethiopia and no exception on compromised water quality of the Ashmaq and Sawur streams attributed to bacterial contamination, eutrophication, sedimentation, and agricultural pollutants. The assessment of water quality status of streams has been a long research experience in the global context^9,14,17,18^.

Over the past decades, countries and organizations worldwide have been developing indicators to identify the anthropogenic stressors’ effect on water quality^5,9,12,18,19^. In Ethiopia, among others, physicochemical and biological indicators have been widely used for determining the natural and anthropogenic influences on water resources and habitat because organisms respond to stresses from multiple spatial and temporal scales^5,16,20^. Accordingly, freshwater macroinvertebrates allow objective classification of the biological quality of aquatic systems with varying degrees of degradation^5,21^. In this regard, aquatic macroinvertebrate species vary in sensitivity to pollution and thus, their relative abundances have been used to make inferences about pollution loads^2,5^. In addition, good water quality resources depend on many physicochemical parameters and the magnitude and source of any pollution load – and to assess that, monitoring of these parameters is essential too^4,6,22^.

Accurate and timely limnological information on water quality is necessary to shape a sound public policy and to implement water safety programs efficiently^6,9,20,23^. Apart from this, the increasing impact of anthropogenic pressure on Ethiopia’s inland water bodies prompted researchers to develop and adopt easy, cheap, holistic and robust water quality assessment tools^4,5,24^. Ashmaq and Sawur streams are among the natural water bodies found in semi-arid central Ethiopia and exposed to intensive human activities that might cause water pollution. Thus, this study was intended to characterize the effect of human activities on the biological and physicochemical profiles of the two streams for sustainable utilization of the resources. In addition, this study was intended to produce baseline limnological information on streams that has been neglected in Ethiopian aquatic research.

Despite the recognized vulnerability of water sources to contamination, there is limited knowledge regarding the water quality status and biological diversity of Ethiopian streams. Therefore, the purpose of this study was to determine the water quality status of Ashmaq and Sawur streams using some water physicochemical parameters and biological indicators. Moreover, the findings of this study will provide valuable insights for policymakers, water resource managers, and stakeholders to formulate effective strategies for water quality management and conservation in the streams of various socioeconomical perspectives. The results are also relevant to frame the agendas and policies of Ethiopia to implement the sustainable development goals (SDGs). The results will also help to improve institutional arrangements towards optimum water resource management and the sustainability of aquatic ecosystems.

## Materials and methods

### Description of the study area and sampling sites

**Fig. 1:**
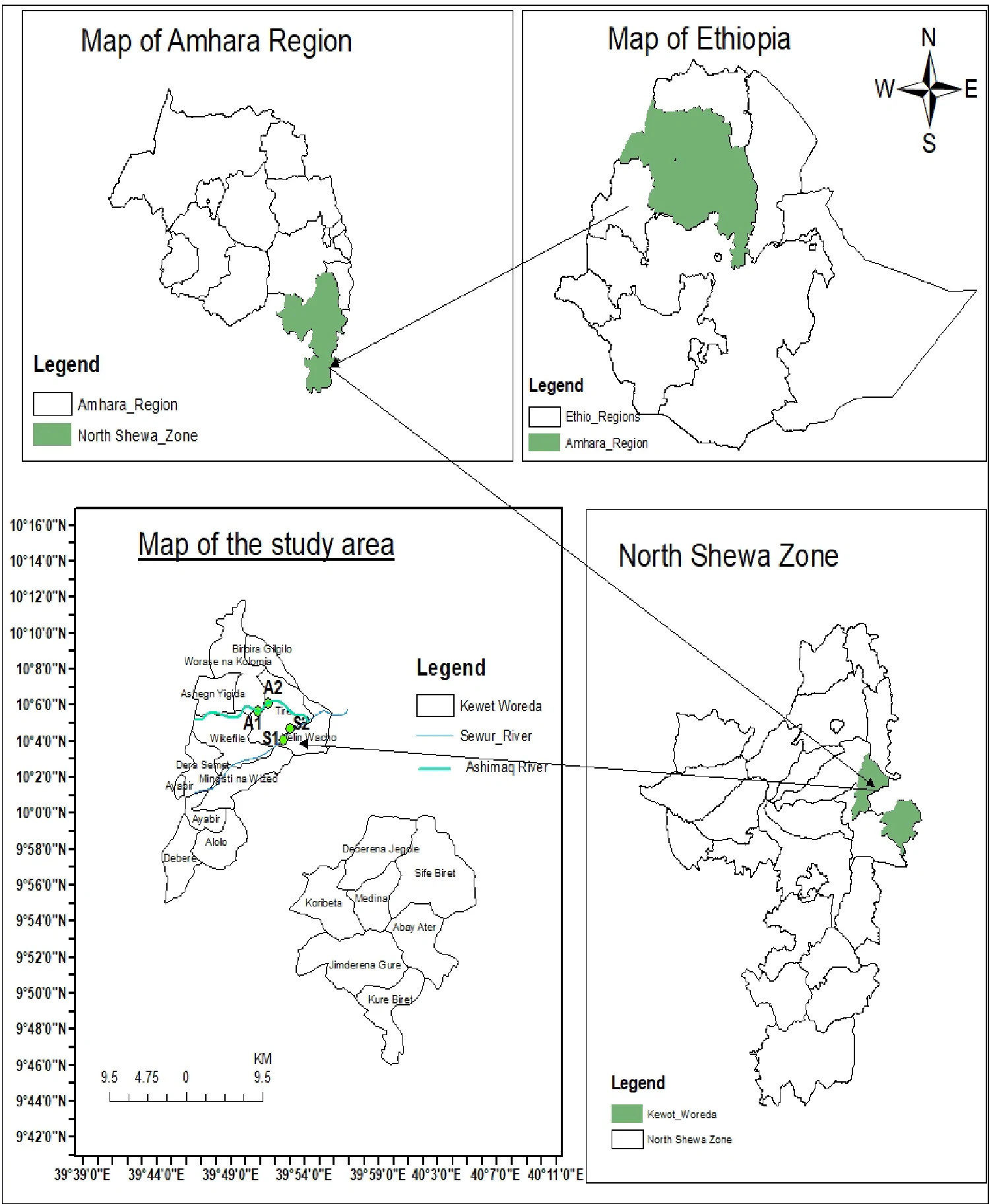
Map of the study area with the sampling localities- A1/A2 and S1/S2 developed using the ArcGIS (the parent map of the country and region is adopted from Amhara Bureau of Agriculture^25^ as adopted from the Central Statistical Agency of Ethiopia).

This study was conducted in Ashmaq and Sawur streams located in Kewot District, Ethiopia. The district is situated at 11^0^55’N and 37^0^20’E and at an altitude of 1380 masl. The area has an average annual rainfall of 1007 mm, with effective rain between June and August and dry from January to April with intermittent pre-rainy months in May. The annual minimum and maximum temperatures of the area are between 16.5 °C and 31^0^ C, respectively. Kewot has an area of 785 km^2^ and the main economic activity in this area is agriculture, which includes dryland farming, irrigation and cattle farming^25^. The nearby areas of the streams are rural and semi-urban, which is largely dominated by agricultural and residential land types. During our observation, considerable proportion of the stream courses are highly exposed to intensive grazing, water abstraction for irrigation, and sand mining. In this study, the general features of Ashmaq and Sawur streams (belongs to the Awash River Basin) were observed from four sampling sites (2 from each stream) (Fig. 1). The Global Positioning System (GPS) readings and other morphometric variables of the sampling sites are presented in Table 1. Sampling sites were selected based on accessibility, bottom substrate, flow rate and level of human interventions. They are designated as A1 (upstream) and A2 (downstream) for Ashmaq and S1 and S2 for Sawur streams.

### Sampling techniques and sample collection

Water, macroinvertebrates and fish samples were taken from April (dry month) and August (wet month) 2022 and 2023 twice during each month or eight times during the entire survey. Samples were taken using sterilized plastic bottles and kept in an ice box for transportation to Biology and Chemistry laboratories of Debre Berhan University (DBU) for analysis. Some physicochemical parameters such as electrical conductivity (EC) (µScm^− 1^), dissolved oxygen (DO) (mgL^− 1^), temperature (^0^C), and pH were measured in-situ using a portable multimeter (model:900P). For these parameters’ measurement, water was sampled at three different depth profiles, and a subsample was prepared from replicated points. In our field activity, stream morphometric variables such as site depth (m) and channel diameter (m) were measured using a rope. Water transparency (cm) using a standard Secchi disc (20 cm) and stream flow rate (cmS^− 1^) were recorded in the field.

Macroinvertebrates were collected using a kick sampling method^26^. The sampling procedure involves the use of a D-frame net with a 500 μm mesh size. The D-frame net was placed on the stream along the stream flow gradient; the substratum upstream was agitated by vigorous kicking and brushing of the D-frame net against the stream bed. Furthermore, large stones and macrophytes were hand-picked and washed into the collecting tray-pan to remove any attached macroinvertebrate as used in Moore & Murphy^26^. This procedure was repeatedly done at each sampling site with a uniform sampling effort of 20 min per sampling site^27^. Then macroinvertebrates were transferred to a 50 ml plastic sampling vial socked in 5% formalin solution for further scrutiny. Fish samples were collected with gillnets and hooks, and the voucher specimens were preserved in 5% formalin to transport to the DBU Biology laboratory for identification.

### Laboratory analysis of water samples

Analytical procedures and techniques used in the analysis of water physicochemical parameters were according to the standard method described by the American Public Health Association- APHA^28^ and Analytical Profile Index (API) freshwater master test kit. The alkalinity of the sample was determined by titrimetric methods^29^. The total hardness of calcium carbonate was estimated by complexometric EDTA titration methods using eriochrome black-T (EBT) indicator, concentration of chloride by volumetric titration (Argent metric/Mohr’s method), total dissolved solid (TDS), total suspended solids (TSS) and total solids (TS) by gravimetric method. The analysis of sulphate (SO_4_^2−^) and phosphate (PO_4_^−3^) was determined by a colorimetric method using a UV-visible Spectrophotometer type (inductively coupled plasma-optical emission Spectrophotometer (ICP-OES model, Perkin Elmer optima 8000). The analysis of NH_3_/NH_4_^+^ and NO_2_^−^ was determined by the method of the API freshwater master test kit.

### Macroinvertebrate and fish identification

In the laboratory, macroinvertebrates were sorted and identified to family level using identification keys^30^. Identified macroinvertebrates were counted and the diversity, number of individuals, and distribution patterns were determined to examine the specific correlation with various water physicochemical variables. Fish was identified to species level using keys^31^.

### Data analysis

Descriptive statistics were used to examine the average values of each physicochemical parameter and to evaluate the relative abundance (%RA) of fish and macroinvertebrates. The macroinvertebrate and fish community structure in relation to environmental variables were assessed using ordination techniques. Detrended correspondence analysis (DCA) was performed and suggest linear species response model and redundancy analysis (RDA) was used for ordination^32^. RDA for a triplot of species, sites and environmental variable data was used to determine community structure^32^. Data were presented as means ± SD and subjected to two-way analysis of variance (ANOVA) for statistical significance at *p* < 0.05. The t-test was performed, followed by pair-wise comparisons to realize the significant difference between each sampling site and months. All statistical analysis and ordination procedures were carried out in PAST version 4.01^33^, SPSS version 20 and Microsoft Office Excel 2013.

In the present study, the Shannon Diversity Index (H’)^34^ and Hilsenhoff Family-level Biotic Indexes (HFBI) were applied to assess species evenness and water quality, respectively. H’ was estimated using the formula: Shannon (H’) = -Σ (pi ln pi); Evenness (e) = H’/lnS; where pi = ni/N, ni = number of individuals of species i, N is the total number of individuals in a sample, and S = total number of species. The HFBI is a method for assessing freshwater stream health based on the tolerance of aquatic macroinvertebrates to organic pollution. A lower HFBI value indicates less pollution, as it signifies a greater abundance of sensitive pollution-intolerant species. HFBI was calculated by HFBI= *∑Xiti*/n^35^; where; Xi is the number of individuals within the taxon, ti is the tolerance value of taxon and n is the total number of organisms in the sample.

### Ethical considerations and consent

Ethical approval was granted by the Department of Biology, Debre Tabor University, on March 14, 2022 (**Ref: DTU/2/Biol-01/2022**), and remained valid for the entire study duration. All animal capturing and laboratory handling procedures adhered to the ethical principles of the Declaration of Helsinki.

**Table 1:** Summary of morphometric variables of the streams.

Sampling Sites	Alt	Site depth (m)		Secchi depth (cm)		Channel diameter (m)		Flow rate (cmS^− 1^)		GPS reading	
		Dry	Wet	Dry	Wet	Dry	Wet	Dry	Wet	*N*	E
A1	1317	0.32 ± 0.09	0.443 ± 0.03	55.00 ± 5.0	5.33 ± 1.53	3.19 ± 0.73	16.00 ± 2.0	13.00 ± 2.0	21.53 ± 2.0	10°18’33”	39°58’13”
A2	1309	0.20 ± 0.03	0.442 ± 0.03	40.00 ± 8.0	9.67 ± 1.53	3.443 ± 1.03	17.33 ± 2.52	7.67 ± 1.53	19.33 ± 1.53	10°06’15”	39°56’18”
S1	1243	0.23 ± 0.07	0.20 ± 0.03	35.00 ± 11.53	9.00 ± 0.5	3.83 ± 0.72	3.93 ± 1.01	12.33 ± 2.08	32.28 ± 2.08	10°17’36”	39°56’38”
S2	1232	0.23 ± 0.06	0.22 ± 0.03	23.67 ± 44.51	11.57 ± 1.68	44.00 ± 0.7	6.00 ± 1.0	12.00 ± 2.0	23.00 ± 2.0	10°18’27”	39°58’52”

Note: Alt.= altitude (m).

## Results

### Stream morphometric variables and physicochemical parameters

The results (mean values) of water physicochemical parameters analyzed from four sampling sites of Ashmaq and Sawur streams across dry and wet months are summarized in Table 2. The stream channel diameter of Ashmaq was different from Sawur in wet months (Table 1), and the difference was statistically significant (*p* < 0.001). Site depth was statistically different at sites between (A1 and S1) and (A2 and S1) both temporally and spatially (*p* < 0.05). However, this parameter was not statistically different between A2 and S2. The highest difference in Secchi depth was recorded at all sites with respect to sampling time, and highest during the dry months. The recorded stream flow rate (cmS^− 1^) was highest at A1 in wet months, and the difference was statistically significant among sites (*p* < 0.05).

The DO concentration was higher in all sampling sites of Ashmaq, while the highest EC was recorded in both months of Sawur streams and in the second sampling site of Ashmaq only during the wet month. High water temperature values were recorded (33.30 ± 0.30 °C) during the dry months at S1 and lower (19.70 ± 2.70 °C) in the wet month at A1 and the difference was statistically significant both in sampling months and sites (*p* < 0.001). During our field expedition, the environmental temperatures of all sites were measured and showed significant variation among sampling sites and months in Sawur stream (*p* < 0.001). The pH measurement in all sites revealed an alkalinity range in both sampling months, except the wet months in S2 where a neutral to alkalinity pH was recorded.

The general trends of the total suspended solids (TSS) and total solids (TS) revealed an increasing pattern during the wet months, and the highest measurement was taken from S1 and S2 during the wet months of these parameters, respectively (Table 2). The total dissolved solids (TDS) which measured 2522.50 ± 31.82 mgL^− 1^ at S2 during the wet month was considerably the highest (Table 2). In the present study, nitrite (NO_2_^−^) levels in samples collected from Ashmaq stream sampling sites varied from 0.00 to 0.22 ppm. All nutrients measured in all streams generally showed an increasing pattern during the wet months. For example, water samples obtained from Sawur stream confirmed the highest NO_2_^−^ concentration and this parameter showed significant difference between Ashmaq and Sawur streams but didn’t show significant variation between S1 and S2 (p ˃ 0.05). The concentration of NH_3_/NH_4_^+^ showed the highest value in sites of Ashmaq streams, but it didn’t show significant variation between sites of Ashmaq streams (Table 2). The concentration of phosphate (PO_4_^2−^) in the study area ranged between 1.63 ± 0.08 and 3.19 ± 1.19 mgL^− 1^, and the difference was statistically significant seasonally, but this parameter didn’t show significant difference among all sampling sites (*p* > 0.05). The lowest and highest values of sulfate (SO_4_^2−^) were recorded at S2 and S1, respectively, and the difference was statistically significant among sites (*p* < 0.05). Unlike the concentration of nutrients, the concentrations of chloride ion (Cl^−^) and almost all measurements of hardness and alkalinity were highest during the dry months. The Cl^−^ was measured along the study sites and ranged between 7.56 ± 1.64 and 34.03 ± 0.0 mgL^− 1^. In the present study, hardness measurement was taken and ranged from 0.28 ± 0.12 to 3.92 ± 0.08 ppm (Table 2). Both Cl^−^ and hardness measurements were not significantly different between the streams sampling sites.

**Table 2:** Summary of descriptive statistical results of water physicochemical variables of the study sites.

Parameters	Sampling sites							
	A1		A2		S1		S2	
	Dry	Wet	Dry	Wet	Dry	Wet	Dry	Wet
DO (mgL^− 1^)	9.65 ± 0.74	7.84 ± 0.05	8.78 ± 0.28	7.87 ± 0.14	6.93 ± 0.30	7.82 ± 0.30	5.96 ± 0.80	8.59 ± 0.53
Temp. (^0^C)	24.00 ± 0.00	19.70 ± 2.70	26.07 ± 0.12	22.60 ± 0.82	33.30 ± 0.30	23.97 ± 0.67	31.7 ± 0.73	23.30 ± 0.87
pH	8.49 ± 0.08	8.83 ± 0.25	8.91 ± 0.04	8.64 ± 0.03	8.44 ± 0.04	8.34 ± 0.66	8.73 ± 0.03	7.44 ± 0.50
EC (µScm^− 1^)	199.53 ± 78.36	402.33 ± 2.52	187.90 ± 61.60	662.00 ± 6.56	642.00 ± 30.05	483.00 ± 4.36	638.00 ± 6.93	419.33 ± 10.02
TSS (mgL^− 1^)	1050.00 ± 70.71	3575.00 ± 106.07	1025.00 ± 55.36	3000.00 ± 0.00	500.00 ± 0.00	3927.50 ± 102.53	2487.50 ± 17.68	2550.00 ± 70.71
TDS (mgL^− 1^)	1512.50 ± 17.68	550.00 ± 70.71	475.00 ± 35.36	600.00 ± 141.42	525.00 ± 35.36	1040.00 ± 56.57	470.00 ± 42.43	2522.50 ± 31.82
TS (mgL^− 1^)	2562.50 ± 88.39	4125.00 ± 176.78	1500.00 ± 0.00	3600.00 ± 141.42	1025.00 ± 35.36	4967.50 ± 45.96	2957.50 ± 60.10	5072.50 ± 102.53
NO^2−^ (ppm)	0.00	0.00	0.22	0.015	0.00	0.21	0.00	0.11
NH_3_/NH_4_^+^ (ppm)	0.55	0.80	0.70	0.90	0.50	0.65	0.26	0.58
PO_4_^2−^ (mgL^− 1^)	1.62 ± 0.08	2.57 ± 0.07	2.56 ± 0.08	3.11 ± 0.54	1.63 ± 0.31	2.82 ± 0.07	3.03 ± 0.02	3.19 ± 1.19
SO_4_^2−^ (mgL^− 1^)	30.99 ± 15.59	55.56 ± 11.11	26.25 ± 0.37	101.16 ± 6.51	195.80 ± 0.56	190.30 ± 7.87	21.29 ± 0.67	44.35 ± 0.75
Cl^−^ (mgL^− 1^)	14.18 ± 2.84	14.18 ± 2.84	15.13 ± 1.64	7.56 ± 1.64	22.03 ± 0.00	21.74 ± 5.90	18.36 ± 0.00	15.13 ± 1.64
Hardness (ppm)	1.68 ± 0.00	0.28 ± 0.12	1.68 ± 0.16	2.20 ± 0.20	3.92 ± 0.08	1.52 ± 0.00	3.61 ± 0.14	1.53 ± 0.06
Alkalinity (ppm)	113.33 ± 20.00	75.56 ± 15.40	111.11 ± 7.70	66.67 ± 13.33	104.44 ± 23.41	113.33 ± 20.00	106.67 ± 0.00	146.67 ± 13.33

Note: DO-dissolved oxygen; temp.-temperature; EC.-conductivity, TSS-total suspended solids, TDS-total dissolved solids, TS-total solids; NO_2_^−^-nitrite; NH_3_-ammonia/NH_4_^+^-ammonium ion; PO_4_^−2^- phosphate; SO_4_^−2^ -Sulphate; Cl^−^chloride).

### Composition of biological communities

#### Macroinvertebrate assemblage and structure

A total of 1386 macroinvertebrates ascribed into three orders and five families were identified during the study period. The Diptera and Hemiptera are represented by two families each. The numerical contribution of identified macroinvertebrates is presented in Table 3. The highest macroinvertebrate diversity (*H*’=1.485) was recorded at A2 of Ashmaq stream, while S1 at Sawur stream contributed relatively small macroinvertebrate diversity (*H’*=1.269) and the macroinvertebrates were more evenly distributed in A2. The HFBI value was determined to detect organic pollution on each site. The value of HFBI ranged from 5.74 to 7.23 with the highest value recorded at Sawur stream (S1) and the lowest value of this index was calculated at Ashmaq stream (A1), revealed that site A1 is relatively good in water quality.

**Table 3:** Number of macroinvertebrates identified from Ashmaq and Sawur streams.

Order		Sampling sites			
	Family	A1	A2	S1	S2
Diptera	Empididae	127	54	120	98
	Chironomidae	71	29	33	32
Ephemeroptera	Baetidae	83	101	20	47
Hemiptera	Vellidae	10	62	18	35
	Corixidae	28	138	156	124
	N	319	384	347	336
	S	5	5	5	5
	H’	1.374	1.485	1.269	1.461
	J’	0.85	0.92	0.79	0.91

Note: N- species abundance, N- species richness.

### Fish communities

A total of 149 fish specimens identified into three species were collected. The identified fish species include *Labeobarbus intermedius*, *Labeo niloticus* and *Garra* Spp. *L. intermedius* and *Garra* Spp. were sampled from Ashmaq stream, while *L. niloticus* was sampled from Sawur stream. The total length of the fish was ranged from 13 to 32.8 cm for *L. intermedius*, 11–18.5 cm for *Garra* Spp., and 12.5–25.6 cm for *L. niloticus*, whereas the total weight was ranged between 96.26 and 135 g for *L. intermedius*, 34 and 56.80 g for *Garra* Spp., and 48 and 78.12 g for *L. niloticus*. The number of individuals collected from Ashmaq stream accounts for 108 specimens. Only 41 specimens were sampled from Sawur stream. Analysis of the relative abundance (%RA) showed that, *L. intermedius* had 51.0% of the overall collection and the remaining 27.5 and 21.5% was contributed by *L. niloticus* and *Garra* Spp, respectively.

### Effects of physicochemical parameters on macroinvertebrates communities in Ashmaq and Sawur streams

**Fig. 2:**
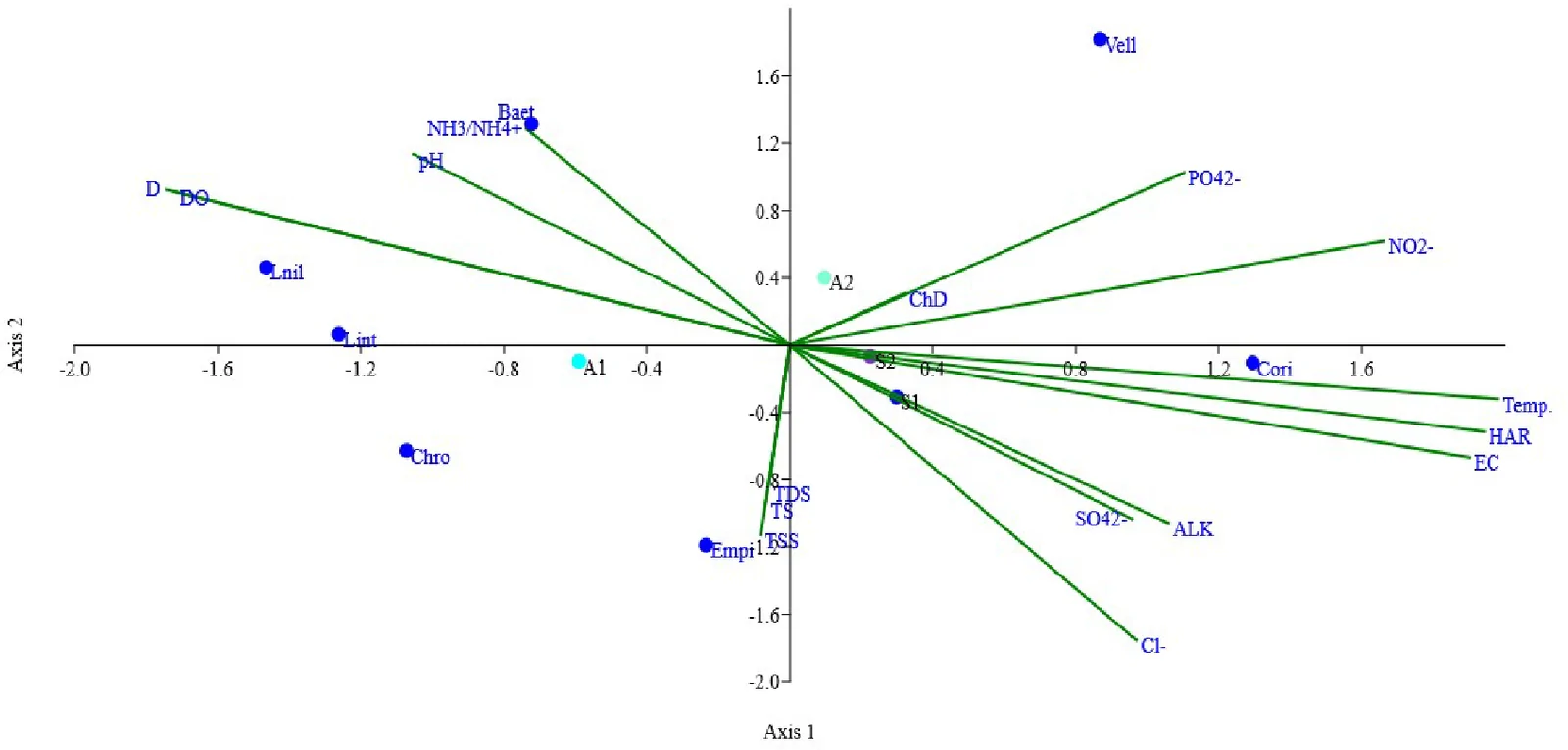
The Redundancy Analysis (RDA) of Species-Environment-Sample triplot showing the relationship of Biological (Macroinvertebrates and Fish) communities (blue shaded circles), environmental variables (green arrow), and sampling points (black shaded circles); acronyms: Depth (D), Channel diameter (Chd), Secchi depth (SD), Hardnes (HAR), Alkalinity (ALK), Baet (Baetidae), Cori (Corixidae), Vell (Vellidae), Chro (Chironomidae), Empi (Empididae); Lnil (*Labeo niloticus*), Lint (*Labeobarbus intermedius*).

The correlation of environmental variables, macroinvertebrate and fish abundance was analyzed using a redundancy analysis (RDA) triplot (Fig. 2). According to the Redundancy Analysis (RDA), water physicochemical parameters such as DO, pH, stream channel diameter, NH_3_/NH_4_ and diversity of organisms like Baetidae and *L. intermedius* were correlated positively with Axis1 and negatively correlated with Axis2 (Fig. 2). In the ordination space, overall, macroinvertebrate and fish assemblages reflected stronger human impact in Sawur, with tolerant taxa dominating, while Ashmaq supported higher diversity and relatively sensitive taxa.

The SO_4_^2−^, NO_2_^−^, transparency (environmental variables) and Vellidae (biological species) correlated positively with Axis1 and Axis2. The diversity of Baetidae and *L. intermedius* largely explained the concentration of DO and stream diameter. In the present analysis, NO_2_^−^ was a major environmental gradient explaining the distribution of Vellidae (Fig. 2). On the other hand, the distribution of Corixidae and *L. niloticus* was mainly explained by EC, PO_4_^−2^, temperature and stream flow rate. The TSS and TDS were the major stream properties that explained the distribution of *Garra* Spp., Chironomidae and Empididae.

## Discussion

### Physicochemical parameters

There was a variation in water physicochemical parameters among sites. The variation may be suggested to the difference in environmental temperature and human activities operated on various stream sections. According to Mezgebu et al.^24^ and Tadesse & Lakew^5^, human activities threaten stream functionality by changing their physical and chemical states. The amount of DO was higher during the dry months. This elevated DO concentration suggests the presence of aeration because of wind action and less human interference for adding anthropogenic waste and thus nearby communities contribute little to organic matter accumulation particularly at Ashmaq stream^5,12^. This stream is relatively protected and has a religious prospect being used by the local communities for decades. It has been reported that the temperature of streams can be governed by the interaction of natural environmental processes (e.g. air temperature, solar radiation, etc.) and anthropogenic disturbances (e.g., irrigation and fishing)^36^. Generally, Ashmaq and Sawur streams’ water temperatures are considerably small compared to criteria set by international organizations.

The pH of aquatic ecosystems is an important indicator of water quality^7,17^. In the present study, the level of pH at Sawur was acidic but slightly alkaline in Ashmaq, which was recorded during the wet months. In agreement with this study, Singh^7^ reported the highest and lowest pH in the dry and wet months, respectively from River Gomti (India). In Awetu river (Ethiopia), Geleta & Firomsa^16^, reported different pH levels along the different sections of this river. The highest pH measured during the dry month may be attributed to the increasing cycle of photosynthetic activity by which solar radiation enhances photosynthesis followed by a rise in pH, leading to a more alkaline situation than the wet month^6,18,23^. On the other hand, the decrease in pH values during the wet month owing to dilution caused by rain may result in CO_2_ liberation from biological oxidation^12,23^. According to WHO^37,44^, an acceptable and suitable water pH is 6.5–8.5 for all aquatic life. Hence, all except, the samples examined at A1 (wet month) and A2 (wet and dry months) and S2 (dry month) were in the acceptable range and safe for healthy aquatic organisms.

The EC recorded from both streams was below the prescribed limit set for drinking purposes^37^. The Food and Agricultural Organization (FAO) has set an acceptable EC of water used for irrigation and the studied streams are suitable for irrigation purposes^38^. In contrast to this study, Jayalakshmi et al.^22^ reported mean of EC values of 225–3350 µScm^− 1^ for the Aiba stream from Nigeria. In Ethiopia, Geleta & Firomsa^16^ reported significantly smaller EC values from the Awetu river located in the Awash basin. The differences in EC of the studied streams with that of the Awetu river could attribute nature of tributaries which carry large particulate matter and human interferences which were large in Ashmaq and Sawur streams.

The mean values of TDS revealed high measurements from all sites. The higher TDS measurement in Ashmaq and Sawur streams suggests elevated agricultural runoff, domestic waste, and washing activities. In line with this study, Jayalakshmi et al.^22^ reported high TDS values (3534 mgL^− 1^) for Labo and Clarin streams in the Philippines. Unlike the present study, Tadesse & Lakew (2024) reported small TDS values (44–111 mgL^− 1^) from the upper Awash River in Ethiopia. All forms of anthropogenic activities including poor agricultural practices and household wastes have been reported to contribute to increasing levels of TDS in Ethiopian streams and rivers^6,13,14,16,24^.

The present study revealed that the TSS was smaller during the dry month. The smaller value in dry months might be because of the decrease in the surface runoff entering streams. Stream water having a TSS value of 100–220 mgL^− 1^ is classified as medium wastewater^39^. Although a more stratified sampling and thorough examination should be considered, based on this study, the TSS value for both streams is higher than the abovementioned reports particularly during the wet months which suggests erosion and runoff from adjacent agricultural lands. In our observation, even if the level of human interference is considerable, it is impossible to say the streams are impacted.

In the wet months, the highest TS was measured in both streams. The primary cause of this elevated TS could be attributed to human activity and surface runoff that enters the stream channel through floods. Consistent with this study, Shirin & Yadav^40^ found that during the rainy month, the highest TS was found in the Ganga Canal water at Haridwar, India. According to Muuz^41^ and Olomukoro et al.^9^, silt and organic matter load are responsible for the existence of large TS. According to the current study, the Cl- concentration which usually indicates sewage content, the values in all the water samples are minor and fall within the acceptable drinking water quality. However, utilization of synthetic agricultural fertilizers needs to be managed as they elevate the concentration of this ion in streams. The WHO recommends that the acceptable limit for Cl- is 250 mgL^[− 1 42^.

Nutrient concentrations in streams are generally highest during the wet months due to increased surface runoff from agricultural and urban areas. This runoff flushes accumulated nutrients from the nearby agricultural lands into the streams. In the present investigation, the NO_2_^−^ levels peaked during the rainy months. This has to do with throwing fertilizer-containing agricultural waste into streams being washed by floods^40^. Similar to this study, Eyayu^11^ found that during the wet month, when agricultural operations are at their highest along river channels, NO_2_^−^ concentrations may rise. However, the NO_2_^−^ concentration in Ashmaq and Sawur streams was below the allowable level established for drinking water^37^. The results obtained from this study showed that high PO_4_^2−^ content was detected in all samples and was above the recommended limit set for drinking water^43^. The increased concentration of PO_4_^2−^ discharged into surface water is usually attributed to agricultural practices, human sewage and use of synthetic detergents^40,44^. The concentration of SO_4_^2−^ in Ashmaq and Sawur streams was between 21.29 ± 0.67 and 195.80 ± 0.56 mgL^− 1^, which is lower than the acceptable limit set for irrigation^38^.

The highest transparency values were recorded during the dry season. This might be associated with the fact that the streams being the recipient of particulate matter or finely divided organic matter and pollutants during flooding can affect turbidity^44,45^. Based on Soni’s et al.^46^ classification, the hardness of Ashmaq and Sawur streams can be categorized as soft water where its hardness level is between 0 and 75 mgL^− 1^.

### Composition of biological communities

#### Macroinvertebrate communities assemblage

Among others, Diptera and Hemiptera were abundant and indicated moderate pollution because they can tolerate fair water quality deterioration. This is further confirmed by the reduction in abundance of Ephemeroptera^30^. According to Salusso & Morana^47^, there are three classes of pollution status based on the H’ of indicator species. By this scale, water bodies with > 3 H′ values mean have no contaminant at all, 1–3 H′, and < 1 H′ values confirming moderate and high pollution levels, respectively. Accordingly, this study found 1–3 H′ values at all sampling sites of both streams and hence moderate water quality. Similar report was made by Popoola & Inwang^48^ in Alaro stream (Nigeria). In contrast to this study, Boudeffa et al.^18^ reported H’ values < 1 and confirming low water quality of Guebli River from Algeria. All sites showed relatively high HFBI values, suggesting the presence of some disturbances^30^. However, a relatively smaller HFBI was calculated at the Ashmaq stream suggesting relatively less anthropogenic disturbance and a high HFBI was estimated at the Sawur stream which suggests a relatively high anthropogenic disturbance. As noted by Hilsenhoff^35^, the HFBI values of 0 to 3.75 indicate excellent water quality. HFBI values of 3.76 to 4.25, 4.26 to 5, 5.01 to 5.75, 5.76 to 6.5, 6.51 to 7.25, and 7.26 to 10 indicate very good, good, fair, fairly poor, poor and very poor water quality, respectively. In this study, therefore, the HFBI value in upstream sites of Ashmaq (A1) represented fair water quality while the values at all the other sites represented poor water quality in relation to HFBI. This suggested that downstream stream courses usually accumulate waste washed from the very headwater regions^18^.

### Fish communities

The diversity and abundance of fish in Ashmaq stream were higher than in Sawur streams and this might be due to the rocky bottom for sheltering the fish species from predators in Ashmaq. This result agrees with the study of Eyayu^11^ who reported higher fish species diversity from Gelegu than Ayima River because of the above-mentioned reason. This might be due to the increasing human interference, and the presence of predation might be attributed to the collection of smaller number of fishes in Sawur stream. Similar to this finding, Welcomme^49^ and Eyayu^11^ observed such variations in the relative abundance of fish population. In the present study, dry season sampling revealed large fish species abundance. This may be associated with the reduction in water volume during the dry season leading to catching more individuals since possible refugees that safeguard fish can dry out. Environmental cues that trigger seasonal fish reproduction might have also contributed to the higher abundance of fish during the dry months^11,50^. According to Welcomme^49^ and Khalid et al.^50^, an abundance of fish in floodplain rivers and streams commonly varies seasonally but increases during the dry season.

## Conclusion

The present investigation reveals that anthropogenic disturbances exert a substantial influence on the stream physicochemical properties. Sites with higher human interference, particularly downstream sites such as S2, showed elevated nutrients, dissolved solids, and electrical conductivity, reflecting compromised water quality. In contrast, the relatively undisturbed upstream sites (A1) demonstrated relatively good water quality as most of the measured physicochemical parameters were in the ranges of standards and dominated by pollution sensitive macroinvertebrate taxa. In the present investigation, the main factors that explained the most significant variance among the biological communities in the streams were DO, pH, site depth, and NH_3_/NH_4_^+^. Although most water physicochemical parameters were found within the permissible limit of international standards, the elevated levels of nutrients and dissolved solids could be indicators compromised stream water quality and stream degradation. It needs to be made aware of the local villagers to safeguard the streams and nearby terrestrial land. Thus, anthropogenic activities that further deteriorate the water quality in Ashmaq and Sawur streams need to be managed through public awareness and institutional arrangements. This includes regular monitoring, land-use regulation, and stakeholder engagement to prevent further degradation. Although, the study of the physicochemical and biological characteristics of streams provides valuable insights into the health and functioning of aquatic ecosystems; however, we are not declared to report a complete data collection procedure regarding inclusions of spatial and temporal sampling and including sample size and sampling frequency. Thus, maintaining a well-managed stream buffer region could support biodiversity and securing the ecological and socio-economic functions of these fragile ecosystems requires a full picture of the streams.

## Data Availability

All datasets and the materials used in the preparation of this manuscript are available from the corresponding author and will be shared upon reasonable request.
